# Field validation of listings of food stores and commercial physical activity establishments from secondary data

**DOI:** 10.1186/1479-5868-5-58

**Published:** 2008-11-10

**Authors:** Catherine Paquet, Mark Daniel, Yan Kestens, Karine Léger, Lise Gauvin

**Affiliations:** 1 School of Health Sciences, the University of South Australia, Adelaide, Australia; 2 Département de médecine sociale et préventive, Université de Montréal, Québec, Canada; 3 Axe santé des populations, Centre de recherche du Centre Hospitalier Universitaire de Montréal, Québec, Canada; 4 Direction de Santé Publique de Montréal, Québec, Canada; 5 Groupe de recherche interdisciplinaire en santé, Université de Montréal, Québec, Canada

## Abstract

**Background:**

Food- and activity-related establishments are increasingly viewed as neighbourhood resources that potentially condition health-related behaviour. The primary objective of the current study was to establish, using ground truthing (on-site verification), the validity of measures of availability of food stores and physical activity establishments that were obtained from commercial database and Internet searches. A secondary objective was to examine differences in validity results according to neighbourhood characteristics and commercial establishment categories.

**Methods:**

Lists of food stores and physical activity-related establishments in 12 census tracts within the Montreal metropolitan region were compiled using a commercial database (n = 171 establishments) and Internet search engines (n = 123 establishments). Ground truthing through field observations was performed to assess the presence of listed establishments and identify those absent. Percentage agreement, sensitivity (proportion of establishments found in the field that were listed), and positive predictive value (proportion of listed establishments found in the field) were calculated and contrasted according to data sources, census tracts characteristics, and establishment categories.

**Results:**

Agreement with field observations was good (0.73) for the commercial list, and moderate (0.60) for the Internet-based list. The commercial list was superior to the Internet-based list for correctly listing establishments present in the field (sensitivity), but slightly inferior in terms of the likelihood that a listed establishment was present in the field (positive predictive value). Agreement was higher for food stores than for activity-related establishments.

**Conclusion:**

Commercial data sources may provide a valid alternative to field observations and could prove a valuable tool in the evaluation of commercial environments relevant to eating behaviour. In contrast, this study did not find strong evidence in support of commercial and Internet data sources to represent neighbourhood opportunities for active lifestyle.

## Background

A growing body of literature supports the association between specific features of neighbourhood built environments, health-related behaviour, and overweight/obesity [1-7]. Relevant to the current obesity epidemic, the presence and density of food and activity-related businesses can provide information about the availability of resources that may support healthful behaviour [8]. A potential alternative to time- and labor-intensive direct observation or surveys of such commercial environments consists of using listings obtained from secondary data sources such as commercial business databases and Internet search engines. These information sources are regularly updated, easily accessible, and are increasingly used in studies investigating the impact of neighbourhood influences on physical activity, eating behaviour, and body mass index [4,7,9-17]. Despite their advantages, questions persist regarding the validity of these data sources as measures of availability of consumer products/resources [18].

In the present paper, we examine the validity of secondary source listings of food stores and commercial physical activity establishments obtained from commercial and Internet information sources. To do so, we draw on the notion of "Ground Truthing" used for validating remotely sensed data through comparison with reference data collected on the ground. In particular, we compare listings derived from the commercial and Internet sources of secondary data with observations conducted in the field for evaluating the utility of such secondary data sources. A secondary aim was to examine whether or not indicators of validity differed according to neighbourhood characteristics and establishment categories.

## Methods

### Overview

Two address listings of food stores and physical activity-related establishments located in 12 census tracts representing the spectrum of socio-economic status (SES) and predominant official Canadian household language (French or English) were developed using a commercial database of businesses and Internet searches. "Ground truthing" was performed by field observations to determine the presence of establishments on each list and to identify establishments absent from lists. Validity statistics were derived from the presence/absence of establishments on lists and in the field.

### Census Tract Sampling

Census tracts were selected from the Montreal Metropolitan Census Area based on 36 socio-demographic variables (2001 Canada Census) chosen for their relevance to research on neighbourhood effects on obesity. These variables were utilised in a Principal Component Analysis for which the first three factors were retained. The first factor was associated with income (e.g., median income and percentage residents below low-income cut-off), the second with ethnic composition (official language (French or English) spoken within households), and the third with education (e.g., percentage residents with a university degree). Factor scores on ethnic composition were used to distinguish dominantly French-speaking (fourth quartile) from dominantly English-speaking (first quartile) census tracts. Education and income factor scores were combined to form a socio-economic index (SEI) weighted more heavily for the education factor (0.70) based on research indicating that education contributes more than income to nutrition-related behaviors and cardiovascular risk factors [19-22]. For each language group, two census tracts were randomly selected within each socio-economic tertile. Two sampled census tracts with high concentrations of an ethnoreligious minority were replaced and alternate selections made randomly. This procedure resulted in six dominantly French-speaking and six dominantly English-speaking census tracts evenly distributed across higher, medium, and lower socio-economic strata (see Additional file 1 for census tracts characteristics).

### Establishments sampled

Two broad categories of commercial establishments relevant to neighbourhood research on obesity were selected: food stores and physical activity-related establishments. Subcategories of food establishments were derived through review of Standard Industry Classification (SIC) codes (provided in the commercial database) and researchers' knowledge of the local commercial environment. SIC is a system for classifying companies and enterprises according to the activities in which they are engaged using 4-digit codes [23]. These codes are complemented with a list of product names, which provide further classification of the individual SIC codes. The following subcategories were chosen for food store establishments: convenience stores (i.e., establishments selling food but no fresh fruits/vegetables), fruit and vegetable stores, specialty markets (e.g., butcher shops, cheese stores), pastry and bakery shops, grocery stores, megamarkets (i.e., very large food stores with large selections of food products), natural food and supplement stores, and small/ethnic markets. We did not consider restaurants and cafés even when takeout was available given our focus on resources available for in-home consumption, nor did we consider retail stores selling food (e.g., WalMart, Dollar Store) given the small proportion of their inventory represented by food items.

We focused on three sub-categories of physical activity-related commercial establishments, namely facilities that offer (1) movement-based activities led by an instructor (e.g., martial arts, yoga), (2) movement-based activities without instruction in motor skills but possibly an activity leader (e.g., fitness centre), and (3) education and coaching (e.g., establishments with trainers, dieticians, or nutritionists). These subcategories were developed by aggregating SIC codes so as to obtain more generic and meaningful subcategories. Non-commercial establishments (e.g., school gymnasiums, parks, playgrounds, outdoor fields, public pools, bike paths) and establishments not directly related to the practice of physical activity (e.g., sport leagues, sport equipment stores) were excluded from this list.

### List development

The commercial list was derived from a commercial database updated in May 2005 [24], wherein establishments were geocoded based on their street address (78%) or postal code (22%). From this database, we extracted commercial establishments located within the 12 census tract boundaries that fell into one of the above subcategories based on their primary SIC code classification, product names and business names (for names of local chains) (see Appendix). A total of 155 food stores and 16 physical activity-related establishments were so identified.

The Internet list was derived from Internet searches conducted in the summer of 2005 with national (, or ) and local (, , or ) search engines, using key words associated with the above subcategories, or names of local chains (see Appendix 1). The search was performed using French websites and French key words since Montreal is a Francophone city and, by law, businesses are required to have French names. We then restricted the list to establishments located within the 12 census tracts boundaries, based on postal code correspondence. Duplicate establishments were eliminated based on names and addresses. Final classification into subcategories was performed post-hoc based on key words and business names. A total of 111 food stores and 12 physical activity-related establishments were so identified.

### Field validation ("ground truthing")

Two observers simultaneously undertook field validation of the commercial (n = 171) and Internet (n = 123) address listings, performed during October 2005 for two weeks within normal business hours. Each observer was responsible for validating one of the two lists. All selected census tract streets were surveyed by foot to verify establishments present in the field. Observers validated establishments based on external cues only. Observers attempted to verify listed establishments based on concordance with at least three of the following characteristics: name, address, category, and subcategory. Establishments found in the field but not present on the original commercial (n = 34) or Internet (n = 69) lists were added to each list. Establishments present on the final lists were classified based on their presence on the initial lists and in field observation.

### Data analysis

To establish concurrent validity, agreement with field observation was assessed for both the commercial and Internet source of listings using percentage agreement computed as the proportion of establishments present on a list and in the field. Although the kappa coefficient is generally preferred to percentage agreement due to its correction for chance agreement, the impossibility of assessing establishments neither observed in the field nor given on either list (resulting in a structural zero [25]) prevented us from generating this statistic. We also computed screening test properties used in epidemiological research, taking field observations as the gold-standard against which both lists were compared. Specifically, sensitivity was obtained from the proportion of establishments found in the field that were present on a list whereas positive predictive value was derived from the proportion of listed establishments found in the field. Other screening test properties (specificity and negative predictive value) could not be calculated due to structural zeros.

Statistics were computed for both lists according to category of establishment, predominant census tract language, and SES tertile. Exact mid-*p *95% confidence intervals were obtained using WinPepi software [26]. We assessed differences between lists, SES tertiles, predominant language group, and establishment category using Fisher's Exact test (two-sided *p*) performed using SAS software (V 9.1, Cary, NC: SAS Institute).

## Results

Validity statistics for both listings of establishments are reported in Additional file 2. For ease of discussion and comparison, we categorised indicators below 0.30 as poor, from 0.31–0.50 as fair, from 0.51–0.70 as moderate, from 0.71–0.90 as good, and over 0.91 as excellent, a categorization that has been previously used [27].

Percentage agreement with field observations was good (range across six SES-language combinations: 0.62–0.78) for establishments identified by the commercial database, and moderate (range: 0.55–0.71) for those identified by Internet sources. The difference in percentage agreement between the lists was statistically significant (Fisher's *p *= 0.006). Within each list, no differences in percentage agreement were found across census tracts according to language or SES tertile (Fisher's *p *> 0.34). However, differences were found across categories of establishments with agreement being higher for food stores than for activity-related establishments for both the commercial (Fisher's *p *= 0.0008) and Internet-based lists (Fisher's *p *= 0.0003).

Overall sensitivity was relatively high (range: 0.67–0.85) for establishments derived from the commercial database and moderate for the Internet-based list (range: 0.55–0.79). Both lists differed in their sensitivity (Fisher's *p *< 0.0001), with correct identification of establishments present in the field being superior for the commercial list compared to the Internet-based list. Sensitivity for individual lists was consistent across predominant language groups and SES tertiles (Fisher's *p *> 0.25), but not across categories of establishments for both the commercial (Fisher's *p *= 0.007) and Internet-based lists (Fisher's *p *= 0.010). Specifically, both lists showed superior sensitivity for food stores compared to physical activity-related businesses. For both lists, most establishments found in the field that were absent from a list (false negative cases) were small-size establishments, such as convenience stores and specialty stores.

Overall positive predictive values were relatively high for both the commercial (range: 0.79–1.00) and Internet-based (range: 0.88–1.00) lists. Chi-square analysis indicated no overall significant differences between lists in terms of positive predictive value (Fisher's *p *= 0.116). For both lists, the positive predictive value did not differ across SES tertiles or language groups (Fisher's *p *> 0.124). Positive predictive value was higher for food establishments than for physical activity businesses for both the commercial (Fisher's *p *= 0.006) and Internet-based lists (Fisher's *p *< 0.0001). Establishments that most often failed on-site validation (false positive cases) included convenience stores, fruit and vegetable stores, education and coaching services, and pastry/bakery shops.

## Discussion

Our data indicate that the concurrent validity of a commercial database was superior to Internet searches as a proxy source of information on food stores and physical activity-related establishments in 12 census tracts. Although the level of correspondence between the commercial list and the observed reality was not perfect, the commercial data source provided a valid alternative to field observations. However, limitations of both commercial and Internet-based data sources should be acknowledged to guide researchers in their use of secondary data and the interpretation of results derived from them.

First, calculations of positive predictive value showed that the commercial list tended to slightly over-represent the availability of food store establishments. Post-hoc examination of field notes revealed that this over-representation was mainly attributable to establishments that were no longer in business (*n *= 20), which casts doubt on the extent to which the commercial database was truly up-to-date. This suggests that researchers seeking to make inferences about the density of food stores could benefit from evaluating via telephone calls whether establishments, especially smaller ones with lower survival rates, are still in operation. Although this strategy has been used on occasion [28,29], it does not appear to be widely implemented.

Sensitivity statistics indicated that the Internet-based list performed less accurately than the commercial list in identifying food source establishments present in the field. This result suggests that Internet sources should be used cautiously in neighbourhood research on food availability, for instance, restricting their use to identify well-defined food stores or restaurant chains [9,10,29] or to supplement listings obtained from other data sources [28,30].

Third, both sources of information were superior in representing food stores compared to physical activity-related establishments, the latter type of establishment being poorly identified by both sources. This poor representation could be due to the wide array of establishment types that could fall within this category, which would complicate efforts to comprehensively assess neighbourhood physical activity opportunities. Studies aimed at capturing neighbourhood potential for active living may circumvent this problem by focusing on either public or specific private opportunities for physical activity [2,12], with only few studies providing comprehensive assessments of physical-activity related commercial establishments [31].

Finally, our results indicate that the performance of neither list varied according to key SES and language groupings by which census tracts can be differentiated in Montreal. This finding, if generalisable, would suggest that studies of food and physical activity establishments identified using proxy measures are not necessarily subject to systematic bias through variation in the validity of proxy measures associated with neighbourhood socio-economic or demographic indicators.

### Study limitations

This study focused exclusively on commercial establishments that can support active lifestyle and offer resources for healthful eating at home. Additional investigations are needed to validate the identification of *restaurants *or take-away outlets by commercial sources, and data sources for *public *spaces used for physical activity. The generalisability of our findings is limited in that we validated measures from just one commercial database. Wang and colleagues [32] have shown that commercial databases vary in their representation of food retail businesses. While a recent U.S. validation study of a different commercial database of physical activity facilities generated estimates very similar to ours [33], future studies might examine multiple commercial databases, where feasible. Although Montréal has certain similarities to other major North-American cities, it is atypical in terms of language composition. Further research is needed to analyse variations in the validity of secondary data sources with neighbourhood characteristics in different cities. In addition, our assessment of the validity of commercial physical activity-related establishments could have been compromised by the lesser numbers of such establishments identified (*n *= 24 for both lists) relative to food stores (*n *= 181 and 168, for commercial and Internet address listings, respectively) within the 12 census tracts sampled and ground-truthed. Our validity estimates are, however, consistent with those reported by Boone and colleagues [33]. Similarly, the precision of estimates for the low-SES census tracts might have been reduced by the lesser numbers of establishments identified for these areas. Finally, although we aimed to provide an exhaustive list of key terms for the Internet search, we acknowledge that our results hinge upon the quality of this list.

## Conclusion

This study represents a first effort to formally assess the validity of secondary data sources pertaining to commercial environments relevant to both healthful eating and active lifestyle, constructs that are increasingly used in research on the built environment and health. Our findings suggest that commercial databases are a valid alternative to expensive field observations in providing a reasonably accurate tool for the identification of available food stores. Commercial and Internet-based data sources both should be used cautiously in representing neighbourhood opportunities for active living.

## Abbreviations

CI: Confidence Interval; SES: Socio-Economic Status; SIC: Standard Industry Classification.

## Competing interests

The authors declare that they have no competing interests.

## Authors' contributions

MD is responsible for communications concerning the manuscript. CP, MD, YK and LG contributed to the study conception and design. YK and MD created the sampling strategy for census tract selection. KL extracted business data from the commercial directory, geocoded business data, and performed data collection in the field. CP conducted statistical analyses. CP and MD interpreted the data. CP wrote the paper. All authors critically revised the article for important intellectual content and approved the final version.

## Appendix

### Internet search terms

Key words for *movement-based activities *were (English translation in parentheses for French key words): conditionnement physique/conditionnement (physical training), musculation, creatine (for muscular training), YMCA, boxe (boxing), gymnastique (gymnastics), sports, golf, tennis, baseball, hockey, basketball, football, athlétisme (athleticism), soccer, natation/nage (swimming), piscine (swimming pool), plongeon (diving), marche/marche à pieds (walking), course (race), marathon, badminton, articles de sports (sports items), entraîneur (coach/trainer), centre culturel (cultural center), organismes sportifs (sports organisation), karate, arts martiaux (martial arts), centre arts martiaux (martial art center), mini-putt, patinage (skating), patinage artistique (figure skating), arena, entraîneur privé (private trainer/coach), gymnase (gymnasium), ballet, ballet-jazz (jazz ballet), claquette (tap dancing), danse (dance), tango, salsa, skate park, conseillers sportifs (sports advisor), triathlon, pentathlon, decathlon, équitation/sports équestres (horseback riding), escalade (climbing), plongée sous-marine (scuba diving), curling, tournois sportifs (sports tournament), lutte (wrestling), escrime (fencing), squash, racquetball, volley-ball, bowling/quilles (bowling), combat.

Key words for *education and coaching establishments *were: alimentation (food), naturiste (naturist), naturopathe (naturopath), naturopathie (naturopathy), obesité (obesity), trouble du comportement alimentaire (eating disorder), anorexie (anorexia), boulimie (bulimia), diététiste (dietician), nutritionnistes (nutritionist), vitamine (vitamin), diète (diet), amincissement/perte de poids (weight loss), kilogramme (kilogram), musculation (muscular training), studio de santé (health studio), équilibre (balance), nutrition.

Key words for *food stores *included alimentation (food), dépanneur (convenience stores), produce market, grocery store, specialty market, grocery, megamarket, supermarché (supermarket), aliments naturels (natural foods), vitamin, herbal products, health food, chaîne d'alimentation (food store chain), épiciers détaillants (food retailer), fruiterie (fruit and vegetable store/market), patisserie (pastry shop), boulangerie (bakery), boucherie (butcher shop/meat market), poissonerie (fish market), marché d'alimentation (food store), fromagerie (cheese shop), pâtes alimentaires (pasta), biscuiterie (cookie/biscuit shop), épicerie fine (gourmet food store), and chains such as Provigo, Marché Métro, Loblaw, Sobeys, IGA, Costco, Axep, Super C, Richelieu, Maxi et cie, Inter-marché, Couche-tard, Dépan-escompte, Dépanneurs 7 Jours.

### Commercial database extractions

For *physical activity establishments*, the following English-only SIC codes and product names (in parentheses) were used:

Professional Sports Clubs And Promoters (Arenas, Baseball Clubs & Instruction, Hockey Clubs & Leagues, Soccer); Physical Fitness Facilities (Gymnastics Clubs, Health & Fitness Program Consultants, Health Fitness & Exercise Services); Membership Sports And Recreation Clubs (Curling Rinks, Golf Courses Private, Recreation Centres, Squash Courts Private, Tennis Courts Private); Amusement And Recreation Nec (Baseball Batting Ranges, Bicycles Renting, Bowling Instruction, Boxing Instruction Clubs, Golf Courses Miniature, Golf Indoor, Golf Instruction, Golf Practice & Driving Ranges, Hockey Instruction, Martial Arts & Self Defense Instruction, Racquetball Courts, Riding Academies, Riding Centres, Rock Climbing, Skating Instruction, Skating Rinks, Ski Clubs, Skiing Instruction, Snow Slides, Swimming Instruction, Swimming Pools Private, Swimming Pools Public, Tennis Instruction, Water Skiing Instruction, Water Slides, Yoga Instruction); Offices Of Health Practitioners Nec (Dietitians & Nutritionists, Nutrition Consultants, Weight Control Services).

For *food stores*, English-only SIC codes and product names were used together with key words for the names of local chains. For each subcategory of food stores, the following SIC code, product name, and key words (where appropriate) were used:

Convenience stores (SIC "grocery store "and product name "convenience store"), Grocery Stores (extraction by SIC "Grocery Stores" and Product Name "Grocers Retail" followed by search for names of local chains), Megamarkets (extraction by SIC major group "food store" followed by search for names of local chains), small/ethnic stores (SIC "Grocery Stores" under product name "grocers retail" not considered as convenience store, grocery stores or megamarkets), fruit and vegetable stores (SIC "Fruit and Vegetable Market"), pastry and bakery shops (SIC "Retail Bakeries"), specialty markets (SIC "Meat And Fish Markets", SIC "Grocery Stores" and Product Name "Gourmet Shops", SIC "Cheese Natural And Processed" and Product Name "Cheese", and SIC "Miscellaneous Food Stores" and Product Name "Poultry Retail").

## Supplementary Material

Additional file 1 **Table 1 – characteristics of Montreal census tracts selected (2001 census data).** Click here for file

Additional file 2 Table 2 – validity statistics for establishments identified using secondary data sources (ground truth observations, Montreal, Canada, 2005). Click here for file

## References

[B1] de Vries SI, Bakker I, van Mechelen W, Hopman-Rock M (2007). Determinants of Activity-friendly Neighborhoods for Children: Results From the SPACE Study. Am J Health Promot.

[B2] Lee C (2007). Environment and Active Living: The Roles of Health Risk and Economic Factors. Am J Health Promot.

[B3] Lee RE, Booth KM, Reese-Smith J, Regan G, Howard HH (2005). The Physical Activity Resource Assessment (PARA) instrument: Evaluating features, amenities and incivilities of physical activity resources in urban neighborhoods [electronic article]. Int J Beh Nutr Phys Act.

[B4] Liu GC, Wilson JS, Qi R, Ying J (2007). Green Neighborhoods, Food Retail and Childhood Overweight: Differences by Population Density. Am J Health Promot.

[B5] Berke EM, Koepsell TD, Moudon AV, Hoskins RE, Larson EB (2007). Association of the built environment with physical activity and obesity in older persons. Am J Public Health.

[B6] Cummins S, Macintyre S (2006). Food environments and obesity – neighbourhood or nation?. Int J Epidemiol.

[B7] Wang MC, Kim S, Gonzalez AA, Macleod KE, Winkleby MA (2007). Socioeconomic and food-related physical characteristics of the neighbourhood environment are associated with body mass index. J Epidemiol Community Health.

[B8] Cohen DA, Scribner RA, Farley TA (2000). A structural model of health behaviour: A pragmatic approach to explain and influence health behaviours at the population level. Prev Med.

[B9] Block JP, Scribner RA, DeSalvo KB (2004). Fast food, race/ethnicity, and income A geographic analysis. Am J Prev Med.

[B10] Cummins SCJ, McKay L, MacIntyre S (2005). McDonald's restaurants and neighborhood deprivation in Scotland and England. Am J Prev Med.

[B11] Pearce J, Blakely T, Witten K, Bartie P (2007). Neighborhood deprivation and access to fast-food retailing a national study. Am J Prev Med.

[B12] King WC, Belle SH, Brach JS, Simkin-Silverman LR, Soska T, Kriska AM (2005). Objective measures of neighborhood environment and physical activity in older women. Am J Prev Med.

[B13] Kipke MD, Iverson E, Moore D, Booker C, Ruelas V, Peters AL, Kaufman F (2007). Food and park environments: neighborhood-level risks for childhood obesity in east Los Angeles. J Adolesc Health.

[B14] Mobley LR, Root ED, Finkelstein EA, Khavjou O, Farris RP, Will JC (2006). Environment, obesity, and cardiovascular disease risk in low-income women. Am J Prev Med.

[B15] Nelson MC, Gordon-Larsen P, Song Y, Popkin BM (2006). Built and social environments associations with adolescent overweight and activity. Am J Prev Med.

[B16] Burdette HL, Whitaker RC (2004). Neighborhood playgrounds, fast food restaurants, and crime: relationships to overweight in low-income preschool children. Prev Med.

[B17] Diez Roux AV, Evenson KR, McGinn AP, Brown DG, Moore L, Brines S, Jacobs DR (2007). Availability of recreational resources and physical activity in adults. Am J Public Health.

[B18] Booth KM, Pinkston MM, Poston WSC (2005). Obesity and the built environment. J Am Diet Assoc.

[B19] Winkleby MA (1992). Socioeconomic status and health: how education, income, and occupation contribute to risk factors for cardiovascular disease. Am J Public Health.

[B20] Popkin BM, Zizza C, Siega-Riz AM (2003). Who is leading the change? U.S. dietary quality comparison between 1965 and 1996. Am J Prev Med.

[B21] Goodman E, Slap GB, Huang B (2003). The public health impact of socioeconomic status on adolescent depression and obesity. Am J Public Health.

[B22] Pill R, Peters TJ, Robling MR (1995). Social class and preventive health behaviour: a British example. J Epidemiol Community Health.

[B23] U.S. Department of Labor Occupational Safety & Health Administration (2008). SIC Division Structure. http://www.osha.gov/pls/imis/sic_manual.html.

[B24] Tamec Inc (2005). Business 411 CD-ROM: May 2005 Update.

[B25] Agresti A (2002). Categorical Data Analysis.

[B26] Abramson JH (2004). WINPEPI (PEPI-for-Windows): Computer programs for epidemiologists [electronic article]. Epidemiol Perspect Innov.

[B27] Janse AJ, Gemke R, Uiterwaal C, Tweel I van der, Kimpen JLL, Sinnema G (2004). Quality of life: Patients and doctors don't always agree: A meta-analysis. J Clin Epidemiol.

[B28] Zenk SN, Schulz AJ, Israel BA, James SA, Bao S, Wilson ML (2005). Neighborhood Racial Composition, Neighborhood Poverty, and the Spatial Accessibility of Supermarkets in Metropolitan Detroit. Am J Public Health.

[B29] Apparicio P, Cloutier M-S, Shearmur R (2007). The case of Montréal's missing food deserts: Evaluation of accessibility to food supermarkets. Int J Health Geogr.

[B30] Glanz K, Sallis JF, Saelens BE, Frank LD (2007). Nutrition Environment Measures Survey in stores (NEMS-S): development and evaluation. Am J Prev Med.

[B31] Gordon-Larsen P, Nelson MC, Page P, Popkin BM (2006). Inequality in the Built Environment Underlies Key Health Disparities in Physical Activity and Obesity. Pediatrics.

[B32] Wang MC, Gonzalez AA, Ritchie LD, Winkleby MA (2006). The neighborhood food environment: sources of historical data on retail food stores. Int J Beh Nutr Phys Act.

[B33] Boone JE, Gordon-Larsen P, Stewart JD, Popkin BM (2008). Validation of a GIS Facilities Database: Quantification and Implications of Error. Ann Epidemiol.

